# Cannabis and the Opioid Crisis

**DOI:** 10.1089/can.2018.29011.rtl

**Published:** 2018-04-01

**Authors:** Daniele Piomelli, Susan Weiss, Graham Boyd, Rosalie Liccardo Pacula, Ziva Cooper

**Affiliations:** ^1^ Department of Anatomy and Neurobiology School of Medicine, University of California, Irvine, Irvine, California.; ^2^ Division of Extramural Research, National Institute on Drug Abuse (NIDA), Rockville, Maryland.; ^3^ New Approach PAC, ACLU Drug Law Reform Project, Washington, DC.; ^4^ BING Center for Health Economics, Drug Policy Research Center, Pardee RAND Graduate School, RAND Corporation, Santa Monica, California.; ^5^ Department of Psychiatry, Columbia University Medical Center, New York, New York.

**On February 9, 2018, the Center for the Study of Cannabis at the University of California, Irvine convened a workshop entitled *Cannabis and the opioid crisis: a multidisciplinary view*. The workshop was held at the Beckmann Center of the National Academy of Sciences in Irvine, California.**

**Participants included Susan Weiss (National Institute on Drug Abuse), Donald Abrams (University of California, San Francisco), Ziva Cooper (Columbia University), Igor Grant (University of California San Diego), Daniele Piomelli (University of California, Irvine), Stanton Glantz (University of California, San Francisco), Marcus Bachhuber (Albert Einstein College of Medicine), Rosalie Pacula (The Rand Institute), Mireille Jacobson (University of California, Irvine), Graham Boyd and Keith Humphreys (Stanford University).**

**Four of the workshop speakers graciously agreed to discuss the topic further and to share their views for the readers of *Cannabis and Cannabinoid Research*. What follows are excerpts from that conversation.**

**Dr. Daniele Piomelli:** The use of opioid drugs has increased dramatically in the United States and so have its two main health consequences, addiction and overdose-related deaths. This is not the first time something like this has happened in the country. The same happened after the Civil War where opiate use rose so much that opioid addiction became known as the “war disease.”

Dr. Susan Weiss, I'd like to ask you: what are the key factors that have brought about the new recent epidemic of opioid misuse and abuse?

**Dr. Susan Weiss:** Thank you, Dr. Piomelli. I think we are all very aware of the tremendous number of overdose deaths that we are seeing now across the country, which has hit almost every region, although certainly some areas more than others. There were more than 65,000 overdose deaths in 2016 and about 50,000 of those were related to opioids.

This crisis began with some well-intentioned attempts to more effectively treat pain, which in turn led to a large increase in the number of opioids being prescribed, up from about 70 million prescriptions in the early 1990s to more than 200 million by 2010.

Among the factors that led to this was a requirement by the Joint Commission on Accreditation of Healthcare Organizations (JCAHO) that physicians assess and more aggressively treat their patients with pain, which included the use of opioids. In addition, physicians were being told that addiction to opioids was very rare in patients with pain, which of course we know now was not correct.

At the same time, there weren't many other options being offered and opioids were thought to treat all sorts of pain, both acute and chronic. Now we know that even that is unlikely to be true—the evidence for effects on chronic pain is very limited, and problems with tolerance (requiring higher doses) and dependence can be difficult to address.

So, as the numbers of prescriptions were rising, resulting in greater environmental availability, our national surveys were starting to show increases in people reporting nonmedical use of opioid medications, most of which were obtained from friends and family members or misuse of their own prescriptions.

At the same time (in the early 2000s) overdose rates started to increase involving prescription opioids. People were becoming addicted—not just pain patients, and in some regions of the country, pill mills and other easy access opioid dispensaries were emerging. Eventually, beginning around 2010, as physicians were becoming more aware of the problem, opioid prescriptions started to go down; “pill mills” were already being closed; and OxyContin, one of the main abused medications, was being marketed in a tamper resistant formulation.

Enter heroin—which was infiltrating more and more of the country, was becoming cheaper, had greater purity, and was easy to get. One could say that there was a perfect storm brewing—prescription opioids were expensive and getting harder to find, people were already addicted, and heroin was readily available.

A study, in fact, in 2015 shows that among people that were in treatment for opioid use disorder, 30% had initiated opioid use with heroin. Thus, there has been a tremendous transition in this country from prescription opioids to heroin use. And, most recently, fentanyl and related synthetic opioids have been wreaking havoc, as these are extraordinarily potent opioid drugs. Deaths from fentanyl and its analogues have now overtaken those from heroin and prescription opioids.

A large spike in deaths in some of the Northeastern States (e.g., New Hampshire and Massachusetts) is attributable to fentanyl, but is also spreading to other parts of the country. And stopping the supply of fentanyl is very challenging because even tiny, tiny amounts can be lethal. In fact, increases in overdose deaths associated with cocaine and methamphetamine may also be related to adulteration with fentanyl.

The opioid epidemic has been evolving. And so, the question of whether cannabis can play a role in abating it is an important one, but we have to keep in mind that we are not talking about the same epidemic that we were even 10 years ago.

The idea that cannabinoids or cannabis may be useful for treating pain and could be a substitute for opioids is an important question for us to consider. There is a significant amount of basic science to support the use of cannabis or cannabinoids—including some of the components of the plant—for their analgesic effects. And, there are some clinical studies that demonstrate meaningful reductions in pain among various patients and many anecdotal reports of people who claim to reduce their opioid dosage or stop using opioids altogether using cannabis.

But we still do not know what forms of cannabis, with what chemical constituents, and at what doses, is being used to treat pain. And none of the clinical trials completed thus far meet the Food and Drug Administration (FDA) standards for approval of a medicine—at least none that use the plant product.

I think we need to be very circumspect in what we are expecting from cannabis with respect to the opioid epidemic. There is no doubt that there are many patients suffering from pain, and we do not have a lot of options to treat it, especially chronic pain. Moreover, the cannabinoid system has a lot of promise regarding analgesic potential and alternative medication approaches. Whether it is the plant, components of the plant, or other strategies to modify endocannabinoid function—these are all possibilities that we need to explore to both help abate the opioid crisis and treat patients with pain who continue to suffer.

**Dr. Piomelli:** Thank you, Dr. Weiss, for this overview. You also have brought us to the very core of the matter at hand today. It does appear that the legal availability of cannabis has consequences on the patterns of opioid use around the country, around the United States and, more in general, on the use of prescription drugs.

There are two important studies by Bradford and Bradford, which were published in 2016^1^ and 2017^2^ in *Health Affairs*. These studies have examined how the behavior of individual physicians changed when medical cannabis laws went into effect in the states that these physicians operated in.

The authors found that medical cannabis laws may be associated with a reduction in the number of prescriptions filled by Medicare and Medicaid. If you look more closely at the data, you see that there are suggestions that opioid analgesics are among the drug classes that are most affected by this decline.

My next question now is for Dr. Rosalie Pacula. You have recently published results that also speak specifically to this problem. Could you tell us about them?

**Dr. Rosalie Liccardo Pacula:** Yes, of course. There is growing evidence examining state-aggregate information on mortality, on treatment admissions, on emergency department data, and even on Medicare and Medicaid claims data, as you just mentioned with the Bradford and Bradford studies that suggest that individuals who are living in states with access to medical cannabis are less likely to have opioid-related prescriptions shown in claims data, emergency room (ER) visits, and treatment admissions shown in Health Care for Uninsured Program (HCUP) and treatment data and then opioid-related mortality as well.

Our recent study in the *Journal of Health Economics* suggested that it is not so much having any medical marijuana law that mattered, that just recognizing the medicinal value of cannabis was not adequate, but it was actually the presence of having active and legally protected dispensaries that increased access to patients and had the significant impact on opioid-related mortality.^3^

In fact, when we conducted replication studies of an earlier study by Marcus Bachhuber and others, we found that just having any medical cannabis law, the impact of that disappeared, and it was only the persistence of open and legally protected dispensaries that mattered for reducing opioid-related mortality.^4^

It was important to note, although, in our study that the impact of the availability of medical cannabis dissipated as more years of data were added to the analysis period. That, we believe, is for one of two reasons. One, the shift in the opioid epidemic, as Dr. Weiss already described might mean that access to medical cannabis is not as relevant for impacting the current opioid epidemic, as people are in fact no longer initiating the opioid use through prescription analgesics.

Or it could be that the newer state policies that adopted these laws protecting dispensaries are more restrictive in their access, because they are more highly regulated. These state policies are changing in response to the 2009 Department of Justice memorandum that advised federal law enforcement not to prioritize enforcement of marijuana laws in states where state policies were more careful in providing clear guidance to the cannabis industry. It is not clear, however, if it is that the more tightened regulations are reducing access or if it is the change in the opioid epidemic that has reduced the link between medical cannabis laws and opioid harm.

**Dr. Piomelli:** That is interesting. What kind of data do you think are needed now and are necessary and sufficient to arrive to a firm conclusion on the issue as to whether, as you pointed out, the legal availability of cannabis products could impact positively the use of opioid drugs? In other words, what study designs are the best to either support or refute this hypothesis?

**Dr. Liccardo Pacula:** Clearly, I think clinical trials, as this would be the gold standard, where you can have randomization of chronic pain patients who take a version of a cannabis product and see what happens to their use and/or need for other opioids versus patients who are given a placebo product that looks like a cannabis product.

In the absence of being able to do that in light of our federal prohibition, there are a couple of states that have adopted policies that require even medical cannabis to be part of prescription drug monitoring programs. In that way, we could document for patients who are using cannabis for medicinal purposes, we could identify who they are. We could identify what opioids they had been prescribed already, as it would be tracked within these prescription drug monitoring programs, and we could see how the prescription drug use (as indicated by prescriptions) evolved.

This would be more of an observational study. It would not be ideal but it could be informative and give us some real guidance as to whether or not what we are seeing in aggregated data is indeed reflective of patient behavior.

**Dr. Weiss:** I would just like to comment on Dr. Pacula's statement above about federal prohibition. While it is true that the federal law—which regards cannabis as a Schedule I substance, with currently only one source of marijuana allowed for research purposes—is certainly a major impediment to doing a randomized clinical trial, it still could be done. There are varying cannabis strains available for research—however, you have to be willing to go through all of the hoops necessary to obtain the Schedule I license and procure the cannabis.

**Dr. Liccardo Pacula:** I agree with that but I think one of the fundamental limitations of the products that can be used for research purposes by the government is that they have much lower levels of the cannabinoid THC than what we are seeing out in the medical markets. And so, it is hard to say that a clinical study that is using the products available through an allowable source, containing substantially lower levels of THC, would be indicative of effects that we would see if they were using one of the other products available in the current legal state markets.

**Dr. Weiss:** That is an important limitation. However, some of the data that were presented at our recent meeting indicated that even with the lower-potency cannabis strains, anti-pain effects could be demonstrated. But I agree with you, we are not able to investigate the diversity of the products available in the dispensaries.

And that is another important question for us—what exactly are people using for pain. Are the products mostly THC? Do they contain other components, such as cannabidiol? How are they taking it? All of those are important considerations if we are to understand whether and how it could benefit people with pain.

**Dr. Liccardo Pacula:** Yes, I agree 100%.

**Dr. Piomelli:** I sense that the conversation is going toward the question as to whether cannabinoids and opioids interact at a pharmacological level. In a recent systematic review of the literature, Bernard Le Foll out of Montreal^5^ has concluded, and I quote, that “Pre-clinical studies provide robust evidence for the opioid-sparing effect of cannabinoids,” which goes back to what Dr. Susan Weiss was saying before. But the same authors, when they delved into the clinical literature, found a much more confusing picture.

Dr. Ziva Cooper, in human laboratory studies just published with Dr. Margaret Haney, reported two important findings.^6^ First, that cannabis may enhance the analgesic effect of the opioid oxycodone, and second, that cannabis may also produce, in her own words, and I am quoting, “Small yet significant increases in oxycodone abuse liability.” These are very important conclusions. Dr. Cooper, could you tell us a little bit more about your findings and about next steps to close the knowledge gap about interactions between opioids and cannabinoids?

**Dr. Ziva Cooper:** Thank you very much for the introduction and for giving a nice synopsis of our study. The objective of the study was to understand, using double-blind, placebo controlled procedures, if cannabis can either substitute for an opioid or if it can produce this opioid-sparing effect where it decreases the effective dose of an opioid to produce an analgesic effect. The hope is that if you decrease the required dose of the opioid needed for pain relief, you will also help to mitigate its adverse effects.

And as Daniele just suggested or just said, there is a great deal of literature from animal laboratory studies demonstrating cannabinoid and opioid synergy for pain, yet this has not yet been explored in a human laboratory until now. For this study, we investigated the effects of smoked placebo cannabis (cannabis without THC), as well as active cannabis (cannabis with THC), in combination with a subthreshold therapeutic dose of oxycodone, a very small dose, 2.5 mg.

Our participants did not have chronic pain, but we measured the impact of cannabis combined with this very low oxycodone dose by eliciting a pain response using a standard pain test. We found significant analgesia, or a decrease in the pain response, when participants smoked the active cannabis in combination with this very low dose of oxycodone. We did not see pain relief in this model with cannabis alone or that dose of oxycodone alone. It was when we put the two together that we saw this “synergistic effect.”

An important aspect of the study is the question related to the possibility that if we observed this cannabis-opioid synergy for pain relief, would we also see an enhancement of some of the adverse effects? One adverse effect that we measured was the impact of the cannabis-opioid combination on abuse liability. We found that the drug combination did not increase cannabis's abuse liability. For example, oxycodone did not increase cannabis self-administration, a gold-standard assessment for a drug's abuse potential. We also found that oxycodone did not increase the positive subjective ratings associated with abuse liability for cannabis, like rating of cannabis's “Good Effect” or how much participants “Liked” the cannabis. Importantly, we did see small increases in abuse liability ratings for oxycodone when combined with cannabis. We did not look at opioid self-administration and that is an important next step.

The study was designed to begin to understand the mechanisms that are driving some of these state level and epidemiological findings that were discussed earlier, as well as the pharmacological interactions to help elucidate can cannabis, or importantly, can cannabinoids in general be used as an adjunct or a substitute for opioids for pain relief.

In future studies, it would be very important to look at the dose-dependent nature of this effect since we only assessed one active cannabis strength. Another research priority is to assess the effects of cannabis that has a variety of other cannabinoids, especially cannabidiol, since there is evidence in the literature that cannabidiol in addition to THC can also help pain. Determining if these findings generalize to a population that has chronic pain under controlled procedures will be fundamental to conclusions related to the clinical utility of cannabis and cannabinoids in combination with opioids to help treat pain.

To respond to the earlier comment regarding the difficulty of studying cannabis and opioid interactions under controlled procedures, there are ways that we can do this. We can look at other FDA approved cannabinoids in a patient population, such as nabilone or dronabinol, in combination with very low doses of opioids to see if we can increase the analgesic effects of opioids, while also mitigating their adverse effects.

**Dr. Piomelli:** What studies do you think are needed now to be able to come to a firm conclusion to this. If you had to have just one ideal study, what do you think would be the strongest one to perform?

**Dr. Cooper:** The strongest study would be a placebo-controlled study with a pain population assessing the impact of cannabis or cannabinoids (and placebo) in combination with low opioid doses. Both the pain-relieving effects and a range of adverse effects would be measured, including abuse liability, cognitive effects, and, importantly, respiratory depressant effects. We do not think that cannabinoids and opioids will interact to enhance the risk of opioid-induced respiratory depression, but it is a critical end-point to address if we want to ensure the safety of opioid–cannabinoid combinations.

This type of study, utilizing a large pain-patient population, is critical in demonstrating the generalizability of our recently published study findings.

And, I think this is feasible. It can be done. It is just a matter of optimizing the study design and figuring out which pharmacological tools to study with respect to both cannabinoid and opioids. For instance, cannabinoid opioid-sparing effects may differ based on the opioid's mu agonist affinity and efficacy. An important question to ask is which opioid agonist would be optimal to administer with cannabis or a cannabinoid to ensure analgesic synergy while also mitigating adverse effects? Do we test a partial agonist like buprenorphine or do we assess the effects of a full agonist?

**Dr. Piomelli:** With respect to your last point, actually, the Le Foll review pointed out that the synergism factor between codeine and cannabinoids and THC is much stronger, 9.5 as opposed to 3.0, for morphine and THC, suggesting that indeed, as you pointed out very directly, the specific opioid matters just as much as the specific dose and type of cannabinoid.

**Dr. Cooper:** Exactly.

**Dr. Piomelli:** So far, we have looked at the cannabinoid–opioid interactions from medical and public health perspective, but we should not forget the societal context, in particular the legal context, which as we all know is extremely confusing.

Graham Boyd, as a lawyer who has been active in this field for some time, can you give our readers the lay of the land from the legal perspective, again on the interactions between the legal status of the two classes of drugs and how that interferes with research and how it can impact also public health?

**Mr. Graham Boyd:** Thank you for that question and lead-in. Cannabis occupies a unique space among the substances deemed to “drugs of abuse.” Conducting medical research on just about any other substance than cannabis is far easier. Within the whole range of opiates that we are concerned, all are prescribed by physicians and can be researched with relative ease, with the exception of heroin, which is in the Schedule I, the most restricted schedule. And that, too, is where cannabis is placed, in Schedule I.

Let me just give a bit of background about this. “Scheduling” is a legal system of classification utilized by the federal government (and mimicked to a large degree by states), and it looks at basically two issues: number 1, the drug's potential for abuse and, number 2, its medical value. If a drug has any potential for abuse, it is put into one of the schedules and if it is a high level of abuse and of no medical value, according to the federal government, then it is put in that most restricted schedule of Schedule I.

And that is where you find cannabis, heroin, and 3,4-methylenedioxymethamphetamine (MDMA or “ecstasy”). And, surprising to most people, the less restrictive Schedule II is where you find cocaine, methamphetamine, and many of the most powerful opiates because those substances do have recognized medical use, despite having a high potential for abuse.

This scheduling paradigm is meant to be based on medicine but, in fact, the initial placement in those schedules for the substances long considered to be “drugs of abuse,” including cannabis, was done in most cases by either the Drug Enforcement Agency (DEA) or Congress and generally not based on thorough review of the science or medicine.

At this point, the overwhelming majority of scientists, medical professionals, and ordinary people agree that cannabis being in Schedule I does not make a lot of sense, and yet efforts to reschedule cannabis have been blocked repeatedly. DEA's own administrative law judge conducted a thorough review of evidence, ruled in favor of rescheduling, but was overruled summarily by the DEA Administrator. After years of litigation, Congressional hearings, and thorough exploration of every conceivable path, DEA's roadblocks led to activists to decide to go directly to state voters to pass the medical marijuana laws through direct ballot measures.

The first medical marijuana law, California's Proposition 215, was the brainchild of longtime activist Dennis Peron, an activist in the ACT UP tradition who had fought against the Reagan Administration's refusal to recognize HIV/AIDS. [https://www.washingtonpost.com/local/obituaries/dennis-peron-a-california-activist-and-father-of-medical-marijuana-died-at-72/2018/01/29/304c9474-050e-11e8-8777-2a059f168dd2_story.html?utm_term=.aa6f5018e1b3] The majority of voters said yes and then in California, medical marijuana was permitted, although it remained illegal under federal law.

Interestingly, the federal government responded by saying marijuana can never be medicine. Donna Shalala, the Secretary of Department of Health and Human Services, was among the Cabinet officials who threatened to arrest any doctor who recommended marijuana to patients in California. I was the lawyer who represented doctors, including Marcus Conant, who was one of the first doctors to diagnose AIDS in a lawsuit that established a First Amendment right to recommend marijuana to patients. [Conant v. Walters, 309 F.3d 629 (9th Cir. 2002), available at https://www.leagle.com/decision/2002938309f3d6291862

This has created an incredibly messy situation in the sense that cannabis is legal under state law, yet illegal under federal law, and retention of cannabis in Schedule I has left it in the place where doing research, as the other speakers have noted, is incredibly hard to do. This creates a Catch-22 because the only way to move something out of Schedule I is based on research and medical evidence and yet the barriers to doing that very research are so strict.

So, that is where we stand right now. In recent years, and I think really because of the proliferation of these state laws which make cannabis so much more accessible, and because of the anecdotal and some research evidence of medical efficacy, the federal government has to some extent loosen the restrictions on doing research.

But it still is, literally, among the hardest substances to do any kind of medical research. You have to get a DEA registration, have security. You have to have a safe or a vault to keep the substance. The DEA visits you to make sure that it is secure enough.

And, at this point, only 20% of cannabis research approved by the federal government is actually even looking at therapeutic use. Eighty percent of it is still looking at abuse, addiction, that sort of thing. And funding for the research is also severely restricted; only 16% of federal funding for cannabis research goes to exploring therapeutic use, including the subjects we are talking about today, the interaction between cannabis and opioids.

So it is a tough position to be in where we were trying to actually see the science move forward and yet because of the legal framework around it, it is so very difficult to do that. Much of the evidence we have right now is anecdotal, very hard to quantify, very hard to really put full credence in, and yet this is the best evidence available as these state laws have changed.

**Dr. Piomelli:** As I was listening to you, Mr. Boyd, I recalled one of the conclusions that the National Academy of Science Panel on the Health Impacts of Cannabis drew last January. It is Conclusion 15.1, which I am reading: “There are specific regulatory barriers, including the classification of cannabis as a Schedule I substance that impedes the advancement of cannabis and cannabinoid research.” This is singled out as the first in a series of research barriers and recommendations issued by the committee.

My question now for the entire group is how do we move forward from here? If this conclusion of the National Academy committee is indeed pertinent, as I think it is, what do we do next?

**Dr. Weiss:** I have a couple of comments. One of the areas that presents a barrier is the current requirement that all cannabis used in research come from a single source, which happens to be the University of Mississippi, supported through a contract with National Institute on Drug Abuse (NIDA). We and others have been advocating for diversification of products and formulations, and the DEA in August, 2016 made a determination that it would be within the legal bounds of the international treaty that governs the control of substances (Single Narcotic Convention) to allow other sources to be licensed to provide cannabis for research purposes.

That could be extremely useful, since it would allow for a diversification of the inventory, and provide a clearer, more direct path for individuals who wanted to develop cannabinoid medications.

My point is that we agree that the research is needed, and the process for obtaining cannabis/cannabinoids should be made easier. Moreover, it would also be helpful for researchers to be able to obtain and analyze samples of what people are purchasing from dispensaries or growing themselves to determine positive or negative health outcomes of real-world products. The current Federal laws prohibit this, so it is a missed opportunity. We are attempting to work with our federal partners to see if there are ways to address that.

I did just want to make one other comment about National Institutes of Health (NIH) funding. There is not a bias against funding good research on the potential therapeutic effects of cannabis or cannabinoids. NIDA's mission is mainly about abuse; however, we are still the largest funder of research to study the basic mechanisms of the cannabinoid system—with the potential to develop therapeutics and the therapeutic effects for pain and treatment of addictions (using cannabidiol, for example). Other NIH institutes fund work that falls within their mission—the National Cancer Institute or the National Institute of Neurological Diseases and Stroke are interested in cancer and epilepsy treatment, respectively. But most of what NIH funds are investigator-initiated research, so if we do not get good proposals, then we can't fund them.

**Mr. Boyd:** I agree with that point, and I think there has also been tremendous movement within the federal government, including the diversification of the supply. That being said, it seems likely that movement will be slow, maybe even glacial, as it has been in years past. That history has made so many people, researchers, as well as activists and advocates, impatient. It really does bring to mind the sort of dynamics that happened around AIDS and HIV medication.

One of the most astonishing recent developments is that Senator Orrin Hatch is now suddenly an advocate for removing the barriers to research. He is somebody who is politically conservative and has antidrug credentials as strong as anyone could have. To me, that is, really where the solution is found is in Congressional action that sweeps those barriers away and lets research proceed in a way that it really should have been all along.

**Dr. Cooper:** The question related to best ways to move this research forward is especially apropos given our political climate right now. And as somebody who is committed to studying both the therapeutic and the adverse effects of cannabis and cannabinoids, I have been dedicated to pursuing this research.

I have thought about different ways to accomplish these goals. One is to find alternate sources of funding, so not necessarily rely on NIDA, because their mission is to study substances of abuse, not necessarily the therapeutic effects. In addition, to look at alternate sources of cannabinoids to understand the therapeutic potential of cannabinoids. For instance, studying different formulations of a cannabinoid-based product to answer some of the questions that are top of mind. Again, this is working within the framework and regulations that we have to deal with right now. I also think a lot about the importance of dissemination of findings. There has been a great deal of interest in the media related to the strong epidemiological and state level findings, promoting research related to the pre-clinical data, as well as controlled human data that will help the public understand the issues related to the clinical utility of cannabis and cannabinoids.

Another important avenue to pushing research forward is to actively reach out to investigators who might not necessarily be directly in the field of cannabis and cannabinoid pharmacology, but who are in clinical areas that are particularly important for understanding the potential therapeutic effects of cannabis and cannabinoids. For instance, collaborating with clinician researchers in oncology, anesthesiology, and neurology would have a significant impact on the quality and generalizability of clinical studies.

Finally, it is important for researchers in our field to understand and work with the regulatory hurdles. To push research forward we must be practiced in working with the FDA, DEA, and working with NIDA when needing access to the cannabis supply. These are just some of the things that we have to do to push research forward.

**Dr. Piomelli:** I would like to throw in my two cents. It is not so much the regulatory hurdles. Those are there and may change, but they are what they are. What concerns me most is the lack of clarity as to what one needs to demonstrate. As Graham Boyd pointed out before, one of the key criteria for a Schedule I drug is the lack of medical uses.

The question is, what does Schedule I define as medical use? In the world of the FDA, we have a clear response to that question. New drug candidates have to go through a series of pre-clinical tests, Investigational New Drug (IND) application, and then clinical trials. At the end of this process, you have your answer: the drug is effective or ineffective, safe or unsafe.

But is this the same path that is required by the DEA to be able to say that? Because if it is, we have that answer already because THC, which is the main, if not the only, truly intoxicating component of cannabis, has gone through those very same studies, those I just mentioned. It has gone through Phase I, Phase II, and Phase III studies. Marinol is an FDA approved drug.

This is where I am confused. There must be other criteria. What are those criteria? Where is it that our researchers need to aim to be able to address the medical value of cannabis? Maybe this is not a fair question to this group. I do not know if anybody has the answer. But maybe we do, so that is why I am raising it.

**Dr. Weiss:** I would like to hear Graham's thoughts but the only thing that I would say is that I do not dispute anything you say, but when there is a petition to reschedule marijuana, the plant, the question immediately becomes what are you talking about. Are you talking about a plant that is mostly THC, that is mostly CBD, that has unspecified different components in it? So you immediately get lost in that morass when you petition to reschedule the plant.

**Dr. Liccardo Pacula:** I would echo the point that I think one of the hurdles about cannabis is the fact that we are trying to schedule a whole plant. We do not schedule the opiate plants, poppy—we do not schedule poppy. We do not schedule coca.

**Dr. Weiss:**—We don't, although some plants have been scheduled based on their main ingredient—for example, Coca leaves are schedule II, because cocaine has an accepted medical use. This was not the case for marijuana and THC, because cannabis was placed in Schedule I legislatively, before dronabinol (synthetic THC) was approved as a medication.

**Dr. Liccardo Pacula:** Okay, I stand corrected. I did not understand that there were any other plants, whole plants that got scheduled, which is a challenge I think for moving forward the medical—the categorization of the products that can come from the plant.

**Dr. Piomelli:** This is where I think the law and the way the law interprets itself is crucial because if indeed, say opium, is Schedule II, then also papaverine is Schedule II, and it is not. Papaverine is a mild relaxant. It is a muscular relaxant that has been used and is still used in therapy, and it is not scheduled.

The problem we see with cannabis is that we have a special situation where not just the plant itself is scheduled but each and every chemical component of the plant that goes under the cannabinoid rubric. You know, in the 1970s, when the Controlled Substance Act was enacted, it could have made sense because at that time we did not know about the existence of cannabinoid receptors, and we did not know that these receptors are responsible for the totality of the effects of THC.

But we know that now. Some of the educational efforts that we scientists should put toward to the public and toward lawmakers are to explain that if there is one substance in cannabis that needs to be perused and needs to be considered carefully, that is THC, because that is the one that intoxicates people. And that particular substance (at least in its synthetic form) is in Schedule III. So anything else that does not intoxicate should not be scheduled at all.

**Dr. Piomelli:** In addition, despite the different scheduling for THC versus its synthetic version, we also know that a synthetic compound that is 99% pure is identical to the plant-derived compound that is 99% pure. That is chemistry 101. But I do not want to belabor the point. Any other comments you would like to add at this point?

**Mr. Boyd:** In trying to make sense of the cannabis plant, its component parts, the various molecules that exist within the plant, whether they are naturally occurring or synthesized and trying to make sense of all of that, you feel like you have gone through the looking glass.

There is not anything that actually makes sense from a scientific point of view, from a legal or regulatory point of view. I think these are all artifacts of a time in which the substances were dealt with from a cultural and political point of view, not from a medical point of view. Trying to unwind all of that piece by careful piece I think really is a fool's errand.

From a legal perspective, either Congress or the DEA has to take action more broadly. In the meantime, one must either pursue the research path winding one's way through this somewhat crazy patchwork maze of regulations to ultimately doing research that is federally approved or, alternatively, one can operate in a way that is protected or allowed by state law, is nominally illegal under federal law, which, for the time being, the federal government is largely not enforcing. Even along this latter path, a fair amount of knowledge is being generated. That is the world we live in right now. It is not one that really makes good sense but it is what is true.

**Dr. Piomelli:** Any other comments? That is a nice close as far as I am concerned but are there any other comments?

**Dr. Cooper:** I guess I will pipe in with a positive forward-looking comment. Even though we are dealing with the immense regulatory hurdles discussed earlier, this is a really exciting time to be involved in this research because there are direct public health implications. The general public is eager to actually understand if this plant provides medical benefits and how can it be best utilized if it does produce therapeutic effects? We are at a time where the public is questioning. They are not just sitting idly back; they are engaging in a conversation with scientists, as well as lawmakers. I do believe this is a pivotal moment in history when we are going to see tremendous growth in research over the next decade.

**Dr. Piomelli:** That is a perfect close. Thank you so much, Ziva. Thank you, Graham. Thank you, Rosalie, Susan, Sophie, everybody. I thought this was terrific. Thank you very much for taking the time to do this.

**Sophie:** Yes, thank you so much and on such short notice. We really appreciate it.

## Abbreviations Used

CBD: cannabidiol
DEA: Drug Enforcement Agency
FDA: Food and Drug Administration
NIDA: National Institute on Drug Abuse
NIH: National Institutes of Health
THC: tetrahydrocannabinol
